# Effect of urban vs. rural residence on the association between atopy and wheeze in Latin America: findings from a case–control analysis

**DOI:** 10.1111/cea.12399

**Published:** 2015-01-27

**Authors:** P Endara, M Vaca, T A E Platts-Mills, L Workman, M E Chico, M L Barreto, L C Rodrigues, P J Cooper

**Affiliations:** 1 Colegio de Ciencias de la Salud, Universidad San Francisco de QuitoQuito, Ecuador; 2 Laboratorio de Investigaciones FEPISQuinindé, Ecuador; 3 Asthma and Allergic Diseases Center, University of VirginiaCharlottesville, VA, USA; 4 Instituto de Saude Coletiva, Universidad Federal da BahiaSalvador, Brazil; 5 Department of Infectious Disease Epidemiology, London School of Hygiene and Tropical MedicineLondon, UK; 6 Centro de investigacion en Enfermedades Infecciosas, Pontificia Universidad Católica del EcuadorQuito, Ecuador; 7 Institute of Infection and Immunity, St George's University of LondonLondon

**Keywords:** atopy, geohelminths, house dust mite, Latin America, rural, tropics, urban, wheeze

## Abstract

**Background:**

The association between atopy and asthma is attenuated in non-affluent populations, an effect that may be explained by childhood infections such as geohelminths.

**Objective:**

To investigate the association between atopy and wheeze in schoolchildren living in urban and rural areas of Ecuador and examine the effects of geohelminths on this association.

**Methods:**

We performed nested case–control studies among comparable populations of schoolchildren living in rural communities and urban neighbourhoods in the Province of Esmeraldas, Ecuador. We detected geohelminths in stool samples, measured recent wheeze and environmental exposures by parental questionnaire, and atopy by specific IgE (sIgE) and skin prick test (SPT) reactivity to aeroallergens.

**Results:**

Atopy, particularly sIgE to house dust mite (HDM), was more strongly associated with recent wheeze in urban than rural schoolchildren: (urban, adj. OR 5.19, 95% CI 3.37–8.00, *P *<* *0.0001; rural, adj. OR 1.81, 95%CI 1.09–2.99, *P *=* *0.02; interaction, *P *<* *0.001). The population fractions of wheeze attributable to atopy were approximately two-fold greater in urban schoolchildren: SPT to any allergen (urban 23.5% vs. rural 10.1%), SPT to HDM (urban 18.5% vs. rural 9.6%), and anti-HDM IgE (urban 26.5% vs. rural 10.5%), while anti-*Ascaris* IgE was related to wheeze in a high proportion of rural (49.7%) and urban (35.4%) children. The association between atopy and recent wheeze was attenuated by markers of geohelminth infections.

**Conclusions:**

Our data suggest that urban residence modifies the association between HDM atopy and recent wheeze, and this effect is explained partly by geohelminth infections.

## Introduction

Atopy is a consistently strong risk factor for asthma in industrialized countries [1, 2], but this association appears to be weaker [3–6] in non-industrialized countries. A predominance of non-atopic over atopic wheeze/asthma has been observed in several Latin American countries including Brazil [3, 4, 7], Peru [8], and Ecuador [9].

The association between atopy and allergic disease appears to be stronger in affluent compared to non-affluent [6] and in urban compared to rural populations [10, 11]. Urban–rural differences in the prevalence of environmental exposures that attenuate atopy could explain these observations. Examples of environmental exposures that may suppress atopy include household pets [12], farming exposures [13, 14], and infections during childhood [15]. Geohelminth infections, that are common chronic parasitic infections of predominantly low-income rural populations in the tropics, may have a role in attenuating the association between atopy and allergic symptoms [16] including wheeze [17, 18].

The aim of this study was to investigate if associations between atopy and recent wheeze are modified by rural vs. urban residence and to explore which environmental exposures including geohelminth infections might contribute to such an effect. To do this, we conducted nested case–control studies comparing schoolchildren living in rural communities, as reported previously [17], with urban neighbourhoods in the Province of Esmeraldas in Ecuador.

## Methods

### Study population and design

Cross-sectional surveys were carried out between March 2005 and January 2010 in schoolchildren living in rural and urban areas in the Province of Esmeraldas, the northernmost province on Ecuador's Pacific coast. The population of Esmeraldas Province in 2010 was 534 092 inhabitants (80% of African descent) of whom 189 504 were located in the Provincial capital, City of Esmeraldas (INEC, 2010). The Province of Esmeraldas is at sea level with an average annual temperature of 28 C and 80% relative humidity. In the City of Esmeraldas, the major sources of income are a port, commercial activities, and an oil refinery, while in the rural area, fishing, subsistence agriculture, logging, and palm oil extraction are the main economic activities. Study neighbourhoods in the City of Esmeraldas were located in the periphery of the city in areas where Afro-Ecuadorian migrants tended to have settled from the rural study Districts in Esmeraldas Province. Further details of the study population and study rationale are provided elsewhere [19].

Two case–control studies were conducted between November 2007 and February 2010, nested within the respective urban and rural cross-sectional surveys. Cases were defined as children with parentally reported recent wheeze (i.e. wheeze in the previous 12 months). Controls were a random sample of those without a history of wheeze ever. At the time of selection of cases and controls, a brief screening questionnaire was administered again to parents to confirm their child's case or control status. Four controls for each case were selected using computer generated random lists. All study measurements were carried out using the same standardized methodologies and materials in both case–control studies by the same field and laboratory observers.

### Questionnaires

Data on risk factors were collected by a questionnaire that was administered in Spanish to the child's parent or guardian and which included the core allergy symptom questions of the ISAAC phase II study [20].

### IgE measurements

Blood samples were collected, and plasma was stored at −20°C until testing. Total IgE and IgE antibodies specific for *Dermatophagoides pteronyssinus, Periplaneta americana* (American cockroach) and *Ascaris lumbricoides* were measured using the Pharmacia CAP system (Phadia AB, Uppsala, Sweden) according to the manufacturer's instructions.

### Allergen skin prick testing

Skin prick testing were performed with seven allergen extracts (Greer laboratories, Lenoir, NC, USA): *Dermatophagoides pteronyssinus/farinae* mix, American cockroach (*Periplaneta americana*), *Alternaria tenuis*, cat, dog, ‘9 southern grass mix’ and ‘New stock fungi mix’, positive histamine, and negative saline controls. A positive reaction was defined as a mean wheal diameter at least 3 mm greater than the saline control 15 min after pricking the allergen onto the forearm.

### Exercise-induced bronchospasm

Each child underwent a vigorous 6-min free running exercise with forced expiratory volume in 1 s (FEV_1_) measured before and 5 min after exercise as described previously [17]. The best of five efforts was used to measure Exercise-induced bronchospasm (EIB): [(Pre-exercise FEV_1_–Post-exercise FEV_1_)/(Pre-exercise FEV_1_)] × 100. EIB was defined as a fall of 10% in FEV_1_ after exercise.

### Stool examinations

Single stool samples were collected and analysed for geohelminth eggs and larvae using the modified Kato Katz and formol-ether concentration methods [21].

### Statistical analysis

In pilot studies, we estimated the prevalence of recent wheeze in the rural and urban areas to be 7.3% and 13.5%, respectively, and that a sample of 4000 children in the rural area and 2500 children in the urban area would be sufficient to yield 200 wheeze cases and 800 non-wheeze controls for the two case–control studies. Such a sample size would have 80% power at *P *<* *0.05 to detect an effect on wheeze of OR<0.6 for common exposures (40–60% prevalence), and OR<0.4 for rare exposures (10%) [19]. Geohelminth infection was defined as the presence of at least one geohelminth parasite in a stool sample. Associations between risk factors and recent wheeze were evaluated by logistic regression, separately for urban and rural studies, with robust standard errors allowing for clustering by neighbourhood or community. Age, gender, and maternal education level were included as *a priori* confounders, and other covariates that were significant in univariate analyses were included also in multivariate models. The associations between atopic markers and recent wheeze were estimated after stratifying urban and rural studies by geohelminth infection status and other key variables. Interaction effects were assessed using Wald tests. Population-attributable fractions (PAFs) were calculated using the formula *P* x (OR-1)/OR where *P* is the prevalence of SPT or allergen-specific IgE among cases**.** Analyses were performed using Stata version 9.

## Results

### Selection of study populations

**Figure 1:**
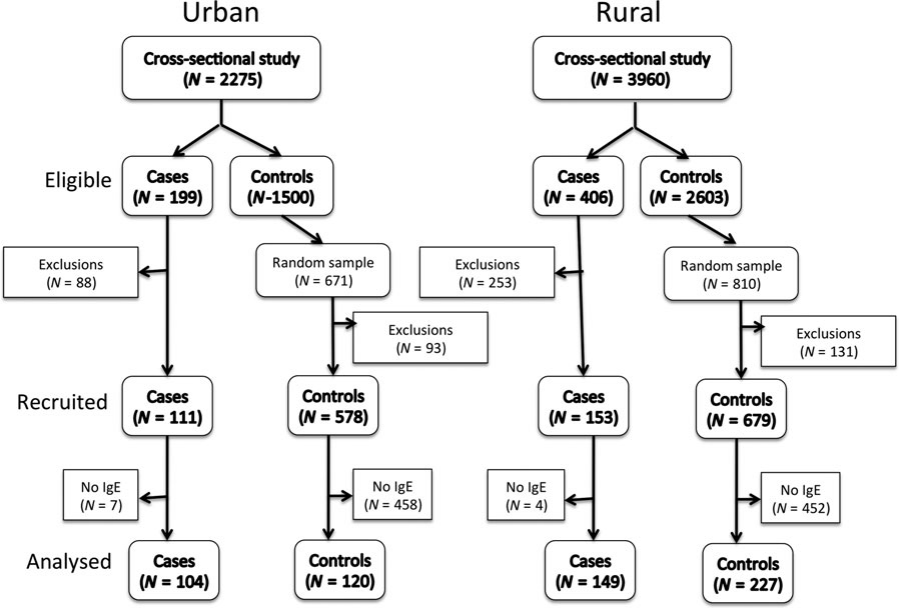
Flow diagram for selection of cases and controls into urban and rural case–controls studies. Eligible cases and controls were those identified as wheezers and non-wheezers in the respective cross-sectional studies who were then evaluated for inclusion in the case–control studies. A random sample of at least four controls was selected for each recruited case. Numbers of recruited cases and controls are shown (Recruited) and those included in the present analysis (Analysed) were those with IgE measurements. Reasons for exclusions from among those eligible were change of wheeze status between cross-sectional and case–control studies, change of address, or unable to contact.

Case–control studies were nested in cross-sectional surveys of 3960 schoolchildren in rural communities and 2275 in urban neighbourhoods of the city of Esmeraldas. The age of study participants ranged from 6 to 19 years. For the present analysis, we included subjects for whom complete data were available for wheeze and allergen-specific IgE from both case–control studies: 149 cases and 227 controls in the rural and 104 cases and 120 controls in the urban case–control study. A flow diagram of selection of subjects for this analysis is provided in Fig.1.

### Factors associated with wheeze

The distributions of risk factors for cases and controls in each of the rural and urban studies are shown in Tables1 and 2, and results of adjusted analyses are shown in Table3. After adjustment for confounders, maternal asthma remained strongly associated with recent wheeze in both study areas. Having a dog living inside the house was a significant risk factor for wheeze only in the urban study (*P *=* *0.03). Recent anthelmintic treatment was associated with an increased risk of wheeze in both studies although this was only significant in the rural area (*P *=* *0.01). The presence of anti-*Ascaris* IgE was a strong risk factor for wheeze in both urban (*P *<* *0.0001) and rural (*P *<* *0.0001) areas. SPT to any allergen was associated with recent wheeze in both areas although the association was stronger in the urban area (urban adj. OR 5.51 [95% CI 1.82–16.60, *P *=* *0.003] vs. rural adj. OR 2.0 [1.05–3.82, *P *=* *0.03]). Similarly, a stronger association was observed between recent wheeze and SPT to HDM in the urban (adj. OR 5.32, 95% CI 2.31–12.25, *P *<* *0.0001) compared to rural (adj. OR 3.07, 95% CI 1.41–6.67, *P *=* *0.005) areas, and there was also a trend of an association between SPT to cockroach and wheeze in the urban but not the rural area. The association between the presence of anti-HDM IgE and wheeze was much stronger in the urban (adj. OR 5.19, 95% CI 3.37–8.00, *P *<* *0.0001) than rural areas (adj. OR 1.81, 95%CI 1.09–2.99, *P *=* *0.02), and a significant interaction by area was observed (*P *=* *0.001).

**Table 1:** Distributions of sociodemographic, hygiene, and other relevant factors between wheeze cases and non-wheeze controls by area of residence. P values were calculated using the χ^2^ test adjusted for clustering. Monthly income represents monthly household income stratified according to the minimum wage of US$250. Recent anthelmintics – anthelmintic treatment in the previous 12 months. Missing values for urban/rural areas were as follows: age (0/2), monthly income (38/8), maternal educational level (5/4), current household smoker (3/3), breastfeeding (21/13), dog inside house (2/0), cat inside house (1/0), and farm animal contact (1/0)

Variable	Rural		*P* value	Urban		*P* value
	Non-wheeze controls *N *=* *227	Wheeze cases *N *=* *149		Non-wheeze controls *N *=* *120	Wheeze cases *N *=* *104	
*Sociodemographics*						
Age (years)						
6–10	56 (24.67)	59 (39.6)		90 (75)	71 (69.61)	
11–19	171 (75.33)	90 (60.4)	0.002	30 (25)	31 (30.39)	0.37
Gender						
Female	99 (43.61)	66 (44.30)		51 (42.50)	49 (47.12)	
Male	128 (56.39)	83 (55.70)	0.85	69 (57.50)	55 (52.88)	0.48
Maternal education level						
Complete secondary	16 (7.21)	14 (9.40)		34 (28.81)	34 (33.33)	
Complete primary	94 (42.34)	55 (36.91)		60 (50.85)	51 (50)	
Illiterate/incomplete primary	112 (50.45)	80 (53.69)	0.53	24 (20.34)	17 (16.67)	0.682
Monthly income						
≤250 USD	160 (76.92)	94 (71.21)		68 (58.62)	56 (56)	
>250 USD	48 (23.08)	38 (28.79)	0.238	48 (41.38)	44 (44)	0.698
Household electric appliances						
0–2	143 (63)	108 (72.48)		42 (35)	52 (50)	
3–4	84 (37)	41 (27.52)	0.058	78 (65)	52 (50)	0.023
*Hygiene exposures*						
Birth order						
0–2	78 (34.4)	59 (39.60)		71 (59.17)	55 (53.40)	
3–4	69 (30.4)	51 (34.23)		31 (25.83)	27 (26.21)	
>4	80 (35.2)	39 (26.17)	0.2	18 (15)	21 (20.39)	0.535
Dog inside house						
No	112 (49.56)	60 (40.54)		91 (75.83)	58 (55.77)	
Yes	114 (50.44)	88 (59.46)	0.08	29 (24.17)	46 (44.23)	0.002
Cat inside house						
No	138 (60.79)	100 (67.57)		73 (60.83)	62 (59.62)	
Yes	89 (39.21)	48 (32.43)	0.176	47 (39.17)	42 (40.38)	0.85
Farm animal contact						
No	155 (68.28)	104 (70.27)		117 (97.50)	99 (95.19)	
Yes	72 (31.72)	44 (29.73)	0.597	3 (2.50)	5 (4.81)	0.353
*Other relevant exposures*						
Breastfeeding						
≤6 months	11 (5.12)	6 (4.26)		4 (3.54)	11 (11.22)	
>6 months	204 (94.88)	135 (95.74)	0.717	109 (96.46)	87 (88.78)	0.03
Current household smoker						
No	143 (63)	94 (63.95)		88 (73.95)	73 (71.57)	
Yes	84 (37)	53 (36.05)	0.85	31 (26.05)	29 (28.43)	0.692
Maternal asthma						
No	186 (86.51)	79 (54.48)		107 (90.68)	56 (55.45)	
Yes	29 (13.49)	66 (45.52)	<0.0001	11 (9.32)	45 (44.55)	0.0001
Recent anthelmintics						
No	117 (52.70)	52 (34.90)		29 (24.37)	14 (13.73)	
Yes	105 (47.30)	97 (65.10)	0.001	90 (75.63)	88 (86.27)	0.046

**Table 2:** Distributions of helminthic infections, atopy markers, and exercise-induced bronchospasm between wheeze cases and non-wheeze controls by area of residence. *P* values were calculated using the χ^2^ test adjusted for clustering. *A. lumbricoides* – infections with *Ascaris lumbricoides*. *T. trichiura* – infections with *Trichuris trichiura*. SPT – allergen skin test reactivity. HDM – to *D. pteronyssinus/D. farinae* mix. Missing values for rural/urban areas were as follows: geohelminth infections (14/7), SPT to any allergen and HDM (3/3), SPT to cockroach (3/4), and exercise-induced bronchospasm (13/3)

	Rural		*P* value	Urban		*P* value
Variable	Non-wheeze controls *N *=* *227	Wheeze cases *N *=* *149		Non-wheeze controls *N *=* *120	Wheeze cases *N *=* *104	
*Helminth markers*						
Any geohelminth infection						
Negative	61 (27.60)	39 (27.46)		72 (61.54)	56 (56)	
Positive	160 (72.40)	103 (72.54)	0.7	45 (38.46)	44 (44)	0.408
*A. lumbricoides*						
Negative	116 (52.49)	77 (54.23)		94 (80.34)	86 (86)	
Positive	105 (47.51)	65 (45.77)	0.8	23 (19.66)	14 (14)	0.269
*T. trichura*						
Negative	92 (41.63)	60 (41.96)		86 (73.50)	61 (61)	
Positive	129 (58.37)	82 (57.75)	0.95	31 (26.50)	39 (39)	0.05
Anti-*Ascaris* IgE						
<0.70 KU/L	97 (42.73)	33 (22.3)		91 (75.83)	51 (49.04)	
≥0.70 KU/L	130 (57.27)	115 (77.7)	< 0.0001	29 (24.17)	53 (50.96)	0.0001
*Atopy*						
SPT to any allergen						
Negative	194 (85.46)	118 (79.73)		110 (91.67)	72 (71.29)	
Positive	33 (14.54)	30 (20.27)	0.147	10 (8.33)	29 (28.71)	< 0.0001
SPT to HDM						
Negative	209 (92.07)	127 (85.81)		112 (93.33)	78 (77.23)	
Positive	18 (7.93)	21 (14.19)	0.05	8 (6.67)	23 (22.77)	0.001
SPT to cockroach						
Negative	212 (93.39)	138 (93.24)		118 (98.33)	93 (93)	
Positive	15 (6.61)	10 (6.76)	0.955	2 (1.67)	7 (7)	0.047
Anti-HDM IgE						
<0.70 KU/L	191 (83.41)	114 (76.51)		107 (89.17)	70 (67.31)	
≥0.70 KU/L	38 (16.59)	34 (22.49)	0.13	13 (10.83)	34 (32.69)	< 0.0001
Anti-cockroach IgE						
<0.70 KU/L	197 (86.03)	122 (81.88)		104 (86.67)	83 (79.81)	
≥0.70 KU/L	31 (13.66)	26 (17.57)	0.30	16 (13.33)	21 (20.19)	0.168
Exercise-induced bronchospasm						
No	213 (96.4)	122 (85.9)		91 (75.8)	70 (69.3)	
Yes	8 (3.6)	20 (14.1)	< 0.0001	29 (24.2)	31 (30.7)	0.28

**Table 3:** Multivariable analysis of factors associated with recent wheeze. PAF% – population-attributable fraction. Interaction *P* value is for interaction by area of residence. *A. lumbricoides* – infections with *Ascaris lumbricoides*. *T. trichiura* – infections with *Trichuris trichiura*. Recent anthelmintics – anthelmintic treatment in the previous 12 months. SPT – allergen skin test reactivity. HDM – to *D. pteronyssinus/D. farinae* mix. Estimates of effect with *P *<* *0.05 are shown in bold. ORs were estimated using logistic regression and were adjusted also for monthly income, number of domestic electric appliances, house construction materials, and frequency of physical exercise. Variables for atopy were excluded from the estimation of the associations between geohelminth infections and wheeze because of possible effect modification

Variable	RURAL			URBAN			Interaction *P* value
	Adjusted OR	*P* value	PAF%	Adjusted OR	*P* value	PAF%	
*Sociodemographics*							
Age (years)							
6–10	1			1			
11–19	0.67 (0.43–1.04)	0.08		1.13 (0.72–1.77)	0.57		0.08
Gender							
Female	1			1			
Male	0.79 (0.49–1.27)	0.34		0.77 (0.46–1.28)	0.32		0.94
*Hygiene exposures*							
Dog inside house							
No	1			1			
Yes	1.49 (0.89–2.49)	0.13		**2.37 (1.09–5.12)**	**0.03**		0.31
Any geohelminth infection							
No							
Yes	1.21 (0.68–2.18)	0.52		1.22 (0.56–2.66)	0.62		0.99
*A. lumbricoides*							
No	1			1			
Yes	1.04 (0.64–1.69)	0.85		0.83 (0.46–1.48)	0.53		0.56
*T. trichura*							
No	1			1			
Yes	1.09 (0.58–2.03)	0.783		1.70 (0.78–3.69)	0.177		0.36
Anti-*Ascaris* IgE							
<0.70 KU/L	1			1			
≥0.70 KU/L	**2.76 (1.61–4.73)**	**<0.0001**	49.7	**3.33 (1.84–6.04)**	**< 0.0001**	35.4	0.64
*Other relevant exposures*							
Maternal asthma							
No	1			1			
Yes	**5.57 (3.25–9.53)**	**<0.0001**		**6.51 (2.66–15.9)**	**< 0.0001**		0.78
Recent anthelmintics							
No	1			1			
Yes	**1.85 (1.14–3.0)**	**0.01**		1.73 (0.94–3.19)	0.08		0.87
*Atopy*							
SPT to any allergen							
No	1			1			
Yes	**2.0 (1.05–3.82)**	**0.03**	10.1	**5.51 (1.82–16.6)**	**0.003**	23.5	0.11
SPT to HDM							
No	1			1			
Yes	**3.07 (1.41–6.67)**	**0.005**	9.6	**5.32 (2.31–12.25)**	**< 0.0001**	18.5	0.36
SPT to cockroach							
No	1			1			
Yes	0.96 (0.32–2.85)	0.95	0.3	4.73 (0.88–25.41)	0.069	5.5	0.09
Anti-HDM IgE							
<0.70 KU/L	1			1			
≥0.70 KU/L	**1.81 (1.09–2.99)**	**0.02**	10.5	**5.19 (3.37–8.0)**	**< 0.0001**	26.5	**0.001**
Anti-cockroach IgE							
<0.70 KU/L	1			1			
≥0.70 KU/L	1.41 (0.72–2.74)	0.31	5.3	1.30 (0.68–2.45)	0.416	4.7	0.86

### Proportion of wheeze attributable to atopy

A high proportion of wheeze was attributable to the presence of anti-*Ascaris* IgE in both studies (rural 49.7% vs. urban 35.4%). A higher proportion of wheeze was explained by atopy in urban compared to rural studies: SPT to any allergen (urban 23.5% vs. rural 10.1%), SPT to HDM (urban 18.5% vs. rural 9.6%), anti-HDM IgE (urban 26.5% vs. rural 10.5%), and any allergen-specific IgE (urban 29.7% rural 13.1%).

### Do environmental exposures explain the stronger association between anti-HDM IgE and recent wheeze among urban children?

Associations between atopy and wheeze were stronger in the urban compared to rural studies, and this effect was particularly marked for anti-HDM IgE for which a significant interaction effect by area was observed (Table3). To try to understand this better, we explored if environmental exposures such as geohelminth infections might be relevant. The findings are provided in Table4 in which associations between recent wheeze and the presence of anti-HDM IgE are shown for each level of environmental exposures of interest with interactions by area of residence. 1) Dogs inside the house – the association appeared to be weaker among children with household dogs but there was no evidence of interaction by area. 2) Recent anthelmintic treatment (i.e. within the previous 12 months) had a much stronger association, but only among urban children (interaction *P *<* *0.001). 3) Monthly income – a stronger effect was seen among urban but not rural children living in households with a monthly income greater than the minimum wage (interaction *P *=* *0.02). 4) Geohelminth infections – the association was stronger in children without active geohelminth infections, and this effect was stronger in urban than rural children (interaction *P *=* *0.04). 5) Anti-*Ascaris* IgE – stronger associations were observed in urban and rural children without anti-*Ascaris* IgE, and among children with *anti-Ascaris* IgE, there was some evidence of a greater association in urban but not rural children (interaction *P *=* *0.03). 6) Stratification into four groups by the presence or absence of the two markers for *Ascaris* infection showed a strong association between wheeze and anti-HDM IgE among children with neither infection marker although there was no statistical evidence of interaction by area.

**Table 4:** Associations between recent wheeze and the presence of anti-HDM IgE stratified by key environmental exposures. Ascaris +/−, presence or absence of *A. lumbricoides* eggs in a stool sample. Anti-*Ascaris* IgE +/−, presence or absence of anti-Ascaris IgE ≥0.7 kU/L. ORs are adjusted for age, gender, maternal educational level, maternal asthma, monthly income (above vs. below the minimum wage) and recent anthelmintic treatment. *P* values are for interaction tests by area of residence. Individual ORs in bold have *P *<* *0.05 – cells too small to allow estimation.

Exposure	ORs for associations between anti-HDM IgE and recent wheeze by relevant exposures		
	Rural OR (95% CI)	Urban OR (95% CI)	Interaction *P* value
Dog inside house			
No	**2.60 (1.11–6.13**)	**3.88 (1.94–7.75)**	**0.39**
Yes	1.22 (0.61–2.43)	–	–
Recent anthelmintic treatment			
No	**2.01 (1.02–3.95)**	1.63 (0.21–12.7)	0.71
Yes	1.46 (0.76–2.82)	**6.18 (4.34–8.79)**	**< 0.0001**
Monthly Income			
≤250 USD	1.73 (0.9–3.31)	**3.24 (1.22–8.53)**	0.27
>250 USD	1.47 (0.48–4.44)	**6.12 (3.45–10.8)**	**0.02**
*Geohelminth exposures*			
Geohelminth infection			
No	**2.62 (1.16–5.91)**	**6.84 (4.72–9.9)**	**0.04**
Yes	1.38 (0.77–2.44)	2.09 (0.38–11.5)	0.75
Anti-*Ascaris* IgE			
<0.70 KU/L	**8.02 (1.37–46.9)**	**3.59 (2.41–5.35)**	0.47
≥0.70 KU/L	1.02 (0.6–1.75)	2.86 (0.6–9.52)	**0.03**
Combinations of variables			
Ascaris −/Anti-*Ascaris* IgE −	**9.33 (2.17–40.0)**	**3.87 (2.18–6.88**)	0.25
Ascaris +/Anti-*Ascaris* IgE −	1.13 (0.18–6.90)	–	–
Ascaris −/Anti-*Ascaris* IgE +	1.26 (0.49–3.20)	**2.92 (1.13–7.52)**	0.20
Ascaris +/Anti-*Ascaris* IgE +	0.79 (0.31–1.97)	2.0 (0.33–11.9)	0.38

## Discussion

We have reported previously the findings of the rural case–control study [17] and now extend these observations to investigate the association between atopy and recent wheeze and the factors that modify this association by comparison with an urban case–control study. The case–control studies were conducted among comparable samples of schoolchildren using the same methodology in urban and rural areas within the same region in tropical Latin America. Our data show that the association between atopy, particularly when measured by the presence of specific IgE to house dust mite (HDM), and recent wheeze is significantly stronger in urban compared to rural children and that this effect may be explained, at least partly, by reduced exposures to or infections with geohelminth parasites in the urban population.

We explored the role of several possible exposures that might explain the apparent dissociation between atopy and wheeze in rural compared to urban populations. Exposures associated with socio-economic development have been suggested to have a role in strengthening the association between atopy and wheeze/asthma [6, 10], a factor that could underlie the increased prevalence of asthma in affluent compared to non-affluent regions [22]. A study of schools serving economically distinct populations in Kumasi, Ghana observed a higher frequency of specific IgE to HDM and also higher antibody titres among affluent cases compared to controls or cases from less-affluent schools [23]. In the present study, there was an association between recent wheeze and anti-HDM IgE among children from more affluent urban households in low-income urban neighbourhoods but not among their more affluent counterparts in the rural population. We also examined the effects on this association of hygiene-associated exposures that were significantly associated with recent wheeze in the adjusted analysis. We observed that the association between anti-HDM IgE and recent wheeze was strongest among: 1) urban children who had received recent anthelmintic treatments; 2) children who were free of active geohelminth particularly in the urban area; 3) children who were negative for anti-*Ascaris* IgE; and 4) children who were negative for both geohelminth infection markers. These data point to an absence of geohelminth infection or of a measurable immune response to geohelminth infections (i.e. anti-Ascaris IgE) as being an important determinant of the strength of the association between HDM atopy and wheeze. Conversely, geohelminth infections or infection markers may contribute to the attenuation of this association. Recent anthelmintic treatment and being free of active geohelminths, in the context of lower rates of exposure in the urban area, may be similar measures for a low risk of infection at any point in time. In contrast in the rural area, where rates of infection are high, recent anthelmintic treatment is generally not associated with geohelminth infection status. Consistent with our study findings, a case–control study of adults in Ethiopia showed a stronger association between SPT to *D. pteronyssinus* and wheeze in urban compared to rural populations despite a higher prevalence of SPT in the rural population, and attributed this effect to the presence of high-intensity parasite infection in the rural population particularly with hookworm [18]. Further, the presence of specific IgE to aeroallergens was associated with rhinitis in uninfected Sri Lankan children, while no association was observed in those with geohelminths [16]. Overall, our data provide further support for the hypothesis that geohelminth infections are responsible, at least partly, for the weaker association observed between atopy and wheeze in some non-affluent populations.

There are several possible explanations for the attenuating effects of geohelminth infections or immunological markers of infection on the association between anti-HDM IgE and recent wheeze. Firstly, geohelminth infections have been consistently associated with a reduced prevalence of SPT [24] although the prevalence of allergen-specific IgE may not be affected [25], indicating that geohelminths may affect the ability of effector cells such as mast cells to respond to an allergen stimulus rather than the process of allergen sensitization. A mechanism by which geohelminth infections might modulate effector cells of the immediate hypersensitivity response is through the induction of immune regulatory cytokines such as IL-10 that are associated with chronic infections with *A. lumbricoides* and *T. trichiura* [7, 26]. Alternatively, the strong attenuating effects of anti-Ascaris IgE observed in the rural population could be explained by immunologic cross-reactivity between Ascaris antigens and HDM. Extensive immunological cross-reactivity has been reported between aeroallergens and helminths including Ascaris [27, 28].

However, in a previous analysis of data from the rural case–control study [17], we surmised that cross-reactivity between HDM and *Ascaris* was unlikely explanation because the effect of anti-Ascaris IgE on wheeze was not altered by adjustment for anti-HDM. Similarly, adjustment for anti-HDM IgE in the urban case–control study did not materially affect the OR for the association between anti-Ascaris IgE and wheeze (data not shown).

Most wheeze was not associated with atopy to aeroallergens in rural and urban case–control studies. The fraction of wheeze attributable to SPT in the rural study was similar to a previous estimate of 11% from a different population in rural Ecuador [9]. However, PAFs observed in urban children were twice those estimated for rural children for all markers of atopy. PAFs for allergen-specific IgE were consistently greater than those for SPT. The PAF for allergen-specific IgE (both HDM and cockroach) in the urban study (23.5%) was similar to that reported (24%) in a study of younger children (range 4–11 years) in urban Brazil [3]. These values, although lower than those reported from affluent countries [24], are consistent with estimates from other non-affluent countries. For example, the ISAAC Phase II study that included 22 countries estimated the PAF for wheeze associated with SPT and allergen-specific IgE to be 40.7% and 45.6%, respectively, in affluent countries, but 20.3% and 18.3%, respectively, in non-affluent countries [9].

A high proportion of wheeze appeared to be explained by the presence of anti-Ascaris IgE in both rural (49.7%) and urban (35.4%) studies, proportions that were somewhat greater than the fractions explained by specific IgE to aeroallergens such as dust mite. A high proportion of wheeze in our study population may be explained by allergic-type responses to the presence of *Ascaris* larvae migrating through the lungs [24]. Such responses may be protective and limit the establishment of infections in the human host but may cause pulmonary inflammation and wheezing illness. It is possible that with declining exposures to geohelminths such as occurs in many urban populations, that allergic responses may become redirected to ubiquitous aeroallergens. A study of children aged 6–12 years in Venezuela [29] provided evidence that anti-*Ascaris* IgE was a risk factor for bronchial hyper-reactivity (BHR) in rural children where geohelminth prevalence was high, while among urban children with a low prevalence of geohelminths, anti-HDM IgE, but not anti-*Ascaris* IgE, was associated with BHR. Similarly, a study of Kenyan children aged 8–13 years showed that in a rural area, SPT to HDM was not associated with exercise induce bronchospasm (EIB), while among urban children it was a risk factor for EIB [30].

Strengths of the study include the measurement of allergen-specific IgE and SPT to the dominant allergens in the study population based on extensive previous studies in Ecuador [6, 9, 17, 31]. We can be confident, therefore, that we detected >90% of atopy by the measurement of specific IgE to house dust mite and cockroach. By repeating the parental questionnaire for inclusion in the case–control study, our wheeze cases were defined by the presence of persistent wheeze, perhaps a more specific marker for asthma illness. Sample sizes for the case–control studies were smaller than originally planned resulting in reduced power to detect associations, particularly for tests of interaction, and we cannot exclude type 2 statistical errors. However, we were able to detect significant associations between atopy and wheeze in urban and rural studies and interaction by study area, the primary study analyses, and hence had sufficient power at least for these. The use of questionnaire data to measure most study exposures may be subject to recall bias. The process of inclusion based on subjects with IgE data could have led to selection bias – however, we believe this unlikely because estimates for the associations between SPT and recent wheeze by study area for the whole case–control samples were not materially different from those obtained for the samples analysed in the present study (data not shown).

In summary, we have analysed data from case–control studies conducted in comparable populations of urban and rural schoolchildren in tropical Latin America and show that the association between IgE sensitization to house dust mite and wheeze is stronger in urban compared to rural populations, and that the attenuation of this association, particularly in rural populations, may be explained by a higher prevalence of geohelminths or IgE sensitization to Ascaris.
